# Contact Mapping Using a Low-Cost Velostat Sensor Array in a Synthetic Ankle Model

**DOI:** 10.3390/s26217057

**Published:** 2026-11-08

**Authors:** Rafael Claramunt, Camilla Laturra, Antonio Ros, Pier Paolo Valentini

**Affiliations:** 1 Mechanical Engineering Department, ETSI Industriales, Universidad Politécnica de Madrid, C/ José Gutiérrez Abascal, 2-28006 Madrid, Spain; antonio.ros@upm.es; 2 Enterprise Engineering Department, Università degli Studi di Roma Tor Vergata, Via del Politecnico, 1-00133 Rome, Italy; camilla.laturra@students.uniroma2.eu (C.L.); valentini@ing.uniroma2.it (P.P.V.)

**Keywords:** flexible pressure sensor, tactile sensor array, ankle contact mechanics, 3D-printed model, Velostat

## Abstract

Estimating joint pressure distribution is crucial for understanding how pathologies disrupt natural biomechanics and for optimizing rehabilitation outcomes. While numerous computational, cadaveric, and “in vivo” studies have investigated joint mechanics, most have focused on the hip and knee, leaving the ankle underrepresented. Flexible matrix sensors offer a compelling solution for intra-articular pressure mapping due to their thin profile and sensing networks. This study presents a single experimental synthetic model of the human ankle joint—characterized by high-curvature joint surfaces—incorporating ligamentous constraints, a custom Velostat™ piezoresistive 6 × 4 sensor array, and low-cost microcontroller electronics. Joint kinematics was evaluated in the sagittal plane across the range of motion from dorsiflexion to plantarflexion in five angular positions under four clinical conditions: intact joint, unreduced posterior malleolar fracture, and fracture fixation using two distinct syndesmotic stabilization techniques. Motion was passively induced without external axial loading to isolate the mechanical contribution of the ligaments. Qualitative intra-articular contact maps revealed distinct differences among surgical approaches. These preliminary results demonstrate the viability of flexible, low-cost piezoresistive sensing systems as a proof of concept for intra-articular contact monitoring in complex anatomical joints.

## 1. Introduction

Knowledge of contact distribution in body joints is essential for understanding how pathologies (bone fractures, ligament injuries) alter the natural joint biomechanics [1,2,3,4,5,6]. These changes can lead to pain, cause improper transfer of loads to other joints and negatively impact both recovery time and overall quality of life.

Theoretical models of whole limbs and others based on the rigid body spring method or applying inverse dynamics have tried to infer the forces and the pressure on several human joints [7,8,9].

Many computational studies [10,11,12,13] have also been conducted, but they entail substantial uncertainties due to non-deterministic characteristics of the biomechanical case analyses, such as the difficulty of replicating realistic boundary conditions and the necessity of simplifying the mechanical properties of biological materials.

Some cadaveric studies [14,15,16,17,18,19,20,21,22,23] and only a few “in vivo” [24,25,26], most of them based on instrumented implants, have been carried out. However, they also present major limitations, mainly in terms of reproducibility.

Physical “ex vivo” simplified models serve as a viable, reproducible alternative to other possibilities, but they also have inherent limitations [20,27].

Most experimental studies are related to the hip and knee, but a few examine the ankle [19,20,21,28,29].

Several biomechanical studies have directly compared ankle trans-syndesmotic screw fixation with suture-button systems under controlled loading conditions. Klitzman et al. demonstrated in a cadaveric model that suture-button fixation maintained syndesmotic reduction while allowing greater sagittal fibular motion than screw fixation [30]. Similarly, Soin et al., using cyclic axial compression and external rotational loading, found broadly comparable fibular motion between suture-button and screw constructs, although screw fixation exhibited a higher failure torque [31]. These findings highlight the distinct mechanical behavior of rigid versus dynamic syndesmotic fixation under controlled loading conditions.

Regarding sensors for joint pressure measurement, the most frequently employed in biomechanics (articulating films, widely used in dentistry) have a long history as qualitative indicators in joints [16,17,28]; only recently have they become semi-quantitative with the aid of computer vision, and strictly under static conditions [19]. With respect to sensors with electrical or optical outputs—and thus fully quantitative—measurements based on instrumented prostheses with diaphragm transducers [15] or capacitive sensors [20] have been utilized. Piezoelectric sensors, widely used for measuring pressure distribution in foot insoles and cushions, are unsuitable for quasi-static scenarios [14]. Even fiber Bragg grating technology has been employed [26]; however, its inherent complexity limits measurements to only a few discrete points.

Matrix sensors have recently become a very attractive option for internal joint pressure measurement due to their thin profile and well-distributed sensing networks [6,18,20,21,22,23]. Unfortunately, commercially available sensors possess limited physical flexibility, which restricts their application on highly curved surfaces. In addition, commercial options offer only a limited selection of shapes and sizes, accompanied by closed hardware and software systems.

The development of inexpensive piezoresistive materials and microcontrollers has recently enabled their use as custom matrices of resistive sensors, allowing simultaneous pressure measurement across multiple closely spaced points for specialized applications [32,33]. Flexible piezoresistive matrices have already been investigated for biomedical pressure mapping. For instance, Kciuk et al. developed a low-cost Velostat™ matrix consisting of a continuous piezoresistive sheet placed between orthogonal conductive rows and columns to form a crossbar architecture [33]. However, their system was designed for pressure mapping on large, substantially planar support surfaces rather than for intra-articular measurements on small, highly curved geometries. More generally, flexible pressure-sensing matrices used in biomedical applications are predominantly based on resistive and capacitive principles, although the existing literature demonstrates considerable heterogeneity in sensor architecture and reported performance characteristics [32].

Thin multiplexed grid-array sensors have also been employed to measure articular contact stresses. Brown et al. reported using specifically designed piezoresistive array sensors to investigate intra-articular contact mechanics following articular fractures [34]. The influence of joint curvature on sensor performance was subsequently addressed by Rudert et al., who developed a dedicated thin-sheet grid-array sensor tailored to the highly curved geometry of the human acetabulum [22]. These studies demonstrate that conformability to complex articular geometries is a critical requirement for intra-articular pressure-sensing systems.

In the ankle, real-time matrix sensors have been used to evaluate tibiotalar contact mechanics under controlled loading conditions. Fitzpatrick et al. evaluated posterior malleolar fractures during motion from plantarflexion to dorsiflexion, demonstrating that joint injury significantly alters the spatial distribution of contact stress [23]. McKinley et al. subsequently showed that articular step-offs can induce transient changes in contact stress and stress gradients that may remain undetected in static measurements alone [6]. The same research group further demonstrated that ankle instability induces greater alterations in contact-stress directional gradients than articular incongruity alone [35], while Tochigi et al. highlighted the contribution of tibiotalar articular geometry to passive ankle stabilization through contact stress redistribution [36].

Despite these previous developments, applying pressure-sensing matrices to small, highly curved articular surfaces remains technically demanding. Therefore, the present study investigates a custom, low-cost Velostat™ crossbar array specifically adapted to the curved surface of the talar trochlea and integrated into a modular synthetic ankle model.

In this work, an experimental study of a physical synthetic model of the human ankle joint—characterized by highly curved articular surfaces—is presented. The model incorporates ligamentous constraints, a custom Velostat™ piezoresistive sensor, and a low-cost commercial microcontroller. Different relative positions between the tibia and talus were evaluated in the sagittal plane across the range of motion from dorsiflexion to plantarflexion under four clinical conditions: intact joint, unreduced malleolar fracture, and the same fracture fixed using two distinct surgical techniques. Motion was passively induced without external axial loading to isolate the mechanical contributions of the ligaments. Intra-articular contact maps were obtained for each case, revealing differences in their shapes when two surgical techniques were employed and qualitatively compared.

Previous studies have demonstrated the potential of various sensing technologies for measuring joint contact mechanics, including piezoresistive, capacitive, and optical systems. However, most reported systems have been developed for relatively accessible or less complex geometries, such as the hip or knee joint, or for externally accessible pressure measurements. Moreover, existing flexible sensor technologies differ considerably in terms of sensing principle, architecture, thickness, flexibility, spatial resolution, measurement range, and implementation complexity, making direct comparisons between systems difficult. For intra-articular applications—particularly in small, highly curved joints such as the ankle—additional challenges arise regarding sensor conformability, integration within restricted joint spaces, and adaptation to complex articular geometries. Therefore, the specific challenge addressed in the present study extends beyond pressure sensing itself to the development of a simple, low-cost, flexible sensing approach capable of mapping relative contact changes across a highly curved tibiotalar interface within a reproducible physical model.

The main contribution of this preliminary proof-of-concept study is the development and application of a low-cost, flexible piezoresistive sensing system tailored to the highly curved articular surface of a synthetic ankle model. The proposed setup integrates a custom 6 × 4 Velostat™ sensor array, microcontroller-based acquisition electronics, and a physical ankle model incorporating simplified ligamentous constraints. This system enables the spatial contact response to be evaluated and compared across different ankle positions and experimental configurations, including the intact joint condition, an unreduced posterior malleolar fracture, and two methods of syndesmotic stabilization. Unlike existing flexible piezoresistive matrix applications—which have been developed primarily for planar or externally accessible surfaces—the present setup embeds the sensing array directly within a small, highly curved intra-articular interface. Consequently, this study demonstrates the feasibility of employing low-cost flexible piezoresistive arrays to assess relative changes in sensor-output distribution within a synthetic ankle model.

## 2. Materials and Methods

A simplified 3D model of a left human ankle joint fabricated from polymeric material was developed for this study (Figure 1).

The geometry of the left foot and ankle was obtained from the ‘Left Foot Bones Anatomical Model’ available on the Printables platform [37]. The model was derived from computed tomography (CT) data and provided at a 1:1 life-size anatomical scale. According to the model documentation, the original dataset was obtained from the NIH 3D Print Exchange and subsequently modified for additive manufacturing by separating the left foot and leg from the original skeleton, remeshing the bone surfaces, adding connections between selected bones, and truncating the tibia and fibula at approximately their distal third. The components used in the present study were manufactured in polylactic acid (PLA) via Fused Deposition Modeling (FDM) using a Creality K2 printer and Creality Print 7.1 software (Shenzhen Creality 3D Technology Co. Ltd., Shenzhen, China). Main printing parameters included a 0.20 mm layer height, two perimeter walls, 10% grid infill, and tree supports with an approximate 30° overhang threshold. No independent quantitative validation of the modified geometry against the original CT dataset was performed.

All bones were printed as rigid solids, as their internal trabecular structure was not critical to the primary objective of this study—namely, evaluating the intra-articular contact distribution between the talus and tibia across multiple foot positions. For the same reason, only the talus and distal portions of the tibia and fibula were fabricated as separate components, whereas the remaining tarsal, metatarsal, and phalangeal bones were printed as a single integrated assembly forming a rigid foot unit.

This rigid foot assembly was secured to a horizontal baseplate, while the remaining three bones (tibia, fibula, and talus) were assembled in their anatomical positions using elastomeric elements simulating key ligamentous structures: the anterior inferior tibiofibular, posterior inferior tibiofibular, anterior talofibular, posterior talofibular, and calcaneofibular ligaments, as well as the four fascicles of the deltoid ligament complex. The tibiofibular interosseous membrane and the constrained articulation between the calcaneus and talus were modeled using soft silicone rubber (PlatSil^®^ FS-20, Polytek Development Corp., Easton, PA, USA) with a Shore A hardness of 22.

Ligamentous constraints were implemented using orthodontic elastic chains attached to retention hooks placed at the anatomical insertion sites of the native ligaments. Attachment sites were centered on relevant anatomical bony landmarks, identified and marked on the 3D-printed models by an experienced orthopedic surgeon prior to hook installation. Specifically, the posterior inferior tibiofibular ligament was reconstructed using five chain segments across an attachment distance of 33.0 mm; the anterior inferior tibiofibular ligament using seven segments over 27.8 mm; the anterior talofibular ligament using 11 segments over 37.4 mm; and the posterior talofibular ligament using four segments over 17.0 mm. The calcaneofibular ligament was reproduced using two parallel strands of three chain segments each, spanning an attachment distance of 28.2 mm. The four components of the deltoid ligament complex were reconstructed individually: the anterior tibiotalar component using seven segments over 24.0 mm, the tibionavicular component using 13 segments over 49.0 mm, the posterior tibiotalar component using five segments over 33.3 mm, and the tibiocalcaneal component using 12 segments over 58.4 mm. Based on the experimentally determined force–displacement response of the orthodontic chains and the elongation imposed by these attachment spans, the estimated initial pretension averaged approximately 6 N.

A rigid metallic shaft was inserted into the tibia portion and served as a reference for relative movement angle between tibia and foot. This shaft was confined in the sagittal plane, and its movement was allowed by means of a pulley system.

Tibial inclination was measured using a BMI160 inertial sensor (Bosch Sensortech GmbH, Reutlinger, Germany) attached to the shaft and connected to the ESP32 through the I^2^C interface. The accelerometer was operated in normal mode with a ±2 g measurement range. The inclination angle θ_x_ about the X axis was estimated directly from the accelerometer components a_y_, a_z_ according to:

θ_x_ = atan2(a_y_, a_z_) × 180/π

No additional digital filtering was applied to the accelerometer signals. The sensor was used to provide a relative angular measurement during the experiments; no independent calibration or experimental accuracy assessment of the BMI160 was performed.

One piezoresistive 0.1 mm thick layer of Velostat^TM^ 1704 series (Desco Industries Inc., Letchworth Garden City, UK) was chosen as sensing material. This layer, made of a polymer with added carbon particles, responds to pressure by decreasing its electrical resistance locally and in a strongly non-linear manner [33]. The total surface covered by the sensing layer was approximately 35 mm × 40 mm.

The piezoresistive sensor was placed between the articulating surfaces of the talus and tibia in a continuous layer of the sensing material. Four copper 0.2 mm diameter wires separated approximately 5.5 mm between them were placed on the talus under the sensor and 6 identical wires perpendicular to the others were also placed between the sensor and the tibial surface. Each geometrical intersecting point between wires constitutes a tactile pixel (taxel) where the electrical resistance (that will change with pressure) is measured. The complete set of taxels is then a 6 × 4 matrix, and the upper side was covered with double-sided sticky tape, as can be seen in Figure 2.

A 3 mm thick neoprene foam tape with a Shore A hardness of 20—falling within the reported range for cartilage according to data of instantaneous modulus of femoral human condyle obtained by [38] when converted into Shore A hardness according to [39]—was placed over the adhesive tape to simulate the viscoelastic behavior of articular cartilage and was lubricated with petroleum jelly at the articulating interface. The adhesive tape serves a dual purpose: it acts as a mechanical support for the neoprene layer and maintains the alignment of the upper conductive wires, while the petroleum jelly infiltrates the open-cell pores of the neoprene foam. This combination of neoprene foam and petroleum jelly acts as a compliance layer, qualitatively reproducing the load-mitigating behavior of natural cartilage.

Orthodontic elastic chains (VIDU VI85306, Creative Dental, Madrid, Spain) were selected as ligament substitutes due to their non-linear tensile response. Uniaxial tensile tests were performed on three orthodontic chain segments in accordance with ISO 21606:2022 using an electromechanical testing machine (250 N load cell; force accuracy Class 0.5 per ISO 7500-1:2018; displacement accuracy Class 1 per ISO 9513:2012) at a constant crosshead speed of 100 mm/min. The mean load–displacement curves with 95% confidence intervals are shown in Figure 3.

Above 6 N, this behavior is qualitatively similar to that reported by Siegler et al. [40] and Attarian et al. [41], although approximately 33% less stiff when compared with the data from [41] for anterior talofibular ligament and calcaneofibular ligament across the 6 N to 20 N range, as determined using the secant modulus method to estimate stiffness. The elastomeric chains were prestretched within the model at the neutral 0° reference position (described in detail below) until reaching the onset of the power-law strain-stiffening regime, thereby mimicking the initial pretension of native ligament conditions but assuming the natural ligament behavior cannot be exactly reproduced because there are not enough reliable experimental comparable data about their actual pretension loads nor are the length, thickness and shape the same between individuals.

The signal acquisition electronics comprised an ESP32 DevKit microcontroller board (Espressif Systems, Shanghai, China) and a 74HC4051 8-channel analog multiplexer/demultiplexer, configured according to the circuit schematic shown in Figure 4.

The ESP32 firmware was developed using the Arduino Integrated Development Environment IDE v2.3.2 (Arduino S.r.l., Monza, Italy).

The system scans a 6 × 4 matrix of 24 Velostat^TM^ pressure-sensing nodes by having the ESP32 sequentially drive six rows (F0–F5). The sensing matrix was powered by the ESP32 3.3 V supply, whose performance was characterized by continuously monitoring it with a Fluke 87V digital multimeter (Fluke Corporation, Everett, WA, USA), showing stability down to the millivolt level for 5 h of continuous operation. Inactive rows were LOW (to reduce crosstalk)—while selecting four columns (C0–C3) via a 74HC4051 multiplexer using two control bits (S0, S1; S2 unused). Each node acts as a variable resistor in series with a 47 kΩ pull-down resistor to form a voltage divider. The multiplexer routes the resulting analog signals to the ESP32 ADC1 (GPIO34), where they are acquired using the Arduino analogRead() function.

The default ADC configuration was used, corresponding to a nominal 12-bit resolution (0–4095 digital counts) and 11 dB attenuation for the original ESP32, with an approximate measurable input-voltage range of 0.15–3.10 V. The effective resolution was not independently characterized.

Before assembling the tibial and ligamentous components, a baseline measurement was acquired with the sensing matrix unloaded on the talar surface. The corresponding readings were stored and subsequently subtracted from the acquired signals to compensate for initial sensor and electronic offsets. Before each experimental trial, the tibia was manually positioned at the neutral 0° position, and the angular reference was reset using the acquisition software. The noise threshold and color-scale settings were maintained constant across the experimental measurements to ensure consistent signal processing and visualization.

A single ADC reading was acquired per sensing element after a 10 μs settling delay following multiplexer selection, without temporal filtering or averaging, with a 20 ms inter-cycle delay providing a nominal scanning rate of approximately 50 Hz. This simplified acquisition strategy was adopted to minimize processing requirements and enable real-time visualization of relative contact patterns, consistent with the qualitative purpose of the experimental setup.

These sequentially measured values are transmitted by the ESP32 microcontroller to a host computer via a serial communication protocol and processed using custom Python software v3.11 (Python Software Foundation, Wilmington, DE, USA) to generate a graphical representation of the voltage distribution as an indicator of joint contact mapping. It should be emphasized that this spatial output does not represent absolute pressure values, as quantitative pressure determination would require a dedicated calibration process. The software applies bilinear interpolation to the discrete voltage measurements to generate two-dimensional voltage maps, as illustrated in Figure 5 alongside the taxel grid overlay and the corresponding color-to-voltage scale.

Several unsuccessful calibration attempts were made to obtain quantitative data by characterizing both the relationship between voltage and pressure and the principal sources of measurement uncertainty (including resolution, drift, zero return, repeatability, hysteresis, inter-taxel homogeneity, cross-talk/phantom effects, and saturation pressure).

Initial calibration trials were conducted by applying controlled compressive loads using a 3 mm diameter cylindrical plunger (flat-ended PLA tipped with neoprene foam) mounted in the same electromechanical testing machine used for evaluating the elastic chains. This small contact diameter was selected to prevent simultaneous indentation across adjacent taxels. However, reliable calibration proved unattainable due to a combination of factors: the inherent viscoelastic behavior of the piezoresistive substrate, highly concentrated contact stresses at the wire intersections, micro-mobility of the underlying talar support, the necessity of removing the double-sided adhesive layer to visually align individual taxels, and the control limits of the testing machine alongside its 1 N force resolution.

Subsequently, another calibration attempt was conducted using deadweight loading; however, the small plunger diameter and micro-mobility of the talar support again rendered the contact unstable, preventing load increments beyond 2 N.

Signal drift was evaluated across three repetitions of a static 1.5 N deadweight load, recorded at 30 s sampling intervals. The response exhibited pronounced viscoelastic behavior during the initial 200 s, followed by reduced signal dispersion from the second test cycle onward. The highly localized contact points at the wire intersections served as the primary driver of signal drift, as they induced elevated local stress concentrations within the Velostat™ layer, pushing the material into a stress regime dominated by its viscoelastic response.

A third attempt was made using dead weights over a matrix of flat Velostat™, where the copper wires were replaced with metallic strips, analyzing two taxels and increasing the loading speed without dwell times. In this case, calibration was partially successful: it showed good repeatability, a fast and full return to zero, and practically no viscoelastic effect, while maintaining the expected non-linear behavior. This suggests that many of the calibration issues observed with copper wires may be related to the highly localized contact, which significantly intensifies contact stresses and, consequently, the viscoelastic effect of the sensing layer when the load is held during the dwell time. Although a more in-depth process should be carried out in the future, metallic strips require more space, making their use on very small surfaces inconvenient.

Consequently, the lack of a formal calibration limits the experimental findings to a qualitative assessment without a direct mapping to absolute pressure units. Nevertheless, within the framework of a comparative study, these qualitative maps provide valuable insights into relative intra-articular contact changes.

Two distinct clinical conditions were evaluated in the experiments: an intact ankle control and a Mason Type 2A posterior malleolar fracture [42] (Figure 6), which corresponds to a Haraguchi Type I fracture morphology [43]. Haraguchi Type I injuries accounted for 67% of cases in the original clinical series reported by Haraguchi et al. [43]. In the physical model, this fracture pattern was accompanied by concomitant disruptions of the fibula, the inferior tibiofibular ligaments, and the distal tibiofibular interosseous membrane.

The posterolateral fragment, involving approximately 20–25% of the tibial plafond surface, was incorporated directly into the 3D-printed tibial geometry as a separate fracture element. The remaining associated injuries were subsequently created on the physical model: a fibular fracture was generated, the distal portion of the interosseous membrane substitute was partially disrupted, and the elastomeric elements representing the anterior and posterior inferior tibiofibular ligaments were removed. For the stabilized configurations, the posterior fragment was manually anatomically reduced against the corresponding tibial fracture bed and secured using a posteroanterior lag screw (Figure 7). In the fractured ankle model, malleolar fixation was performed—simulating clinical surgical practice—using a posteroanterior cannulated screw (4 mm outer diameter, 28 mm length, partially threaded; Smith & Nephew plc, Watford, UK; applied tightening torque of 1 N·m). The concomitant fibular fracture was stabilized using a metallic plate (Posterior Distal Tibia 3HL, 63 mm; Smith & Nephew plc, Watford, UK) secured with non-surgical hardware (3 mm × 10 mm screws; applied tightening torque of 1 N·m).

Fracture reduction was verified by direct visual inspection of the fracture margins to confirm anatomical restoration of the tibial articular surface continuity; no independent quantitative imaging-based verification was performed.

The unreduced fracture state and two distinct methods of distal tibiofibular syndesmotic stabilization were evaluated: a trans-syndesmotic screw, which provides rigid construct fixation between the tibia and fibula, and a flexible suture-button construct (comprising a high-strength polymeric suture spanning metallic buttons), which accommodates physiological micromotion between the bones (Figure 8).

The experimental protocol evaluated four experimental conditions representing key clinical states: (1) intact ankle control; (2) unreduced Mason Type 2A posterior malleolar fracture; (3) Mason Type 2A fracture with osteosynthesis (lag screw and fibular plate) combined with rigid trans-syndesmotic screw fixation; and (4) Mason Type 2A fracture with osteosynthesis combined with flexible suture-button syndesmotic stabilization. For the trans-syndesmotic screw configuration, a 3.5 mm × 25 mm cortical screw was inserted and tightened to a torque of 1 N·m. For the flexible stabilization configuration, an INVISIKNOT Ankle Syndesmosis Repair Kit (Smith & Nephew plc) was deployed. The system comprises two stainless-steel metallic buttons connected by a high-strength ultra-high-molecular-weight polyethylene (UHMWPE) suture. A trans-syndesmotic tunnel was drilled through the distal fibula and tibia using the 3.5 mm passing pin provided with the kit. The suture-button construct was subsequently passed through the bone tunnel, resting the medial button against the medial tibial cortex and the lateral button against the lateral fibular plate. The suture was manually tensioned until clinical stabilization of the distal tibiofibular syndesmosis was achieved. This construct provided a compliant, flexible connection between the tibia and fibula, contrasting with the rigid constraint imposed by the trans-syndesmotic screw.

The testing procedure sequence consisted of driving motion in the sagittal plane, continuously and without stopping, from 30° of plantarflexion to 17° of dorsiflexion (Figure 9).

The three positions on the right are representative of relative tibiotalar kinematics during typical human gait phases, alongside two hyperextended extreme joint positions.

No external axial load was applied to the joint construct; motion was driven manually via the pulley system while monitoring elapsed time with a chronometer (total duration from 30° plantarflexion to 17° dorsiflexion: approximately 1 min). Consequently, the internal forces generated by the passive ligamentous constraints represented the sole external force inputs acting across the joint interface.

Three repetitions of each entire experiment were made to qualitatively verify the repeatability of the contact voltage distribution maps, which were continuously recorded. Between repetitions, no change was made to the setup, nor were the ligament substitutes detached and reattached.

One series of three repetitions was carried out for each of the four surgical approaches simulated. The foot, sensor and neoprene layer were always the same for the four surgical simulations. The only change between them was the tibia-fibula assembly.

The maps for the selected angles were extracted from the continuous record of only one of the three repetitions to compare the four surgical approaches.

## 3. Results

For the intact joint control condition (1), the two-dimensional relative contact voltage distribution maps corresponding to the five evaluated joint articulation angles are illustrated in Figure 10.

The corresponding relative contact voltage distribution maps for the unreduced fracture condition (2) across the five evaluated joint positions are presented in Figure 11.

For the rigid fixation condition (3), the distal tibiofibular syndesmosis was stabilized using a trans-syndesmotic screw, completely constraining relative motion between the tibia and fibula. The corresponding relative contact voltage distribution maps across the five evaluated joint positions are illustrated in Figure 12.

Finally, for the flexible stabilization condition (4), the distal tibiofibular syndesmosis was stabilized using a flexible suture-button construct, accommodating controlled physiological micromotion between the tibia and fibula. The corresponding relative contact voltage distribution maps across the five evaluated joint positions are illustrated in Figure 13.

## 4. Discussion

It must first be emphasized that the total force generated across the joint varies with each foot position due to differences in passive ligamentous tension. Direct comparisons of absolute peak voltage magnitudes across different sagittal angles are therefore invalid; evaluation must instead focus on the relative spatial distributions within each contact map.

For the intact control condition (1), the voltage distribution during the plantarflexion phase is concentrated primarily in the central region, accompanied by a secondary peak localized at the posterolateral corner. In the neutral position, the voltage maxima shift anteriorly toward the medial and lateral margins while maintaining the posterolateral peak. As articulation progresses into dorsiflexion, the contact distribution migrates further anteriorly—with the complete resolution of the posterolateral peak—and shifts predominantly toward the peripheral medial and lateral regions.

In the unreduced fracture condition (2), disruption of the posterolateral fragment renders the corresponding articular surface non-load-bearing, eliminating detectable contact in this region. Joint contact shifts predominantly to the medial aspect across all joint angles, deviating markedly from the physiological patterns observed in condition (1) and producing pronounced local voltage concentrations at the extreme limits of plantarflexion and dorsiflexion.

When comparing the intact control condition (1) with the rigid fixation state (3), several notable localized alterations emerge in condition (3), despite broad similarities in overall spatial distribution. In position A, a pronounced voltage peak develops at the posteromedial corner; this peak persists in position B with reduced intensity before completely resolving from position C onward. In positions C, D, and E, lateral contact shifts further anteriorly and displays higher relative intensity compared to condition (1). Furthermore, position E exhibits a localized voltage peak at the anteromedial corner that was absent in the intact control (1).

Conversely, comparing the intact control condition (1) with the flexible stabilization state (4) reveals a substantial overall reduction in contact voltage magnitude across condition (4). This attenuation drew attention immediately, and the direct observation of the joint showed joint incongruity and clear tibiotalar gap formation, as illustrated in Figure 14.

Fernández-Rojas et al. [44] underscored that the posterior malleolus contributes not only to the tibiotalar load-bearing surface but also to distal tibiofibular syndesmotic stability, which directly aligns with the alterations in contact distribution observed in the present study when comparing conditions (1) and (2). These authors further emphasized the critical clinical necessity of restoring anatomical joint congruity. Additionally, Alonso-Rasgado et al. [45] demonstrated that non-physiological contact distribution shifts compressive loads onto cartilage regions that are typically subjected to lower mechanical stress, thereby accelerating focal cartilage degeneration and contributing to the etiology of post-traumatic osteoarthritis.

From a biomechanical perspective, although the contact distribution in the rigid screw fixation condition (3) exhibits less spatial similarity to the intact joint control, it maintains load-bearing contact and avoids the severe contact attenuation observed in the flexible suture-button condition (4).

These findings are qualitatively consistent with previous biomechanical investigations demonstrating distinct mechanical behavior between rigid screw fixation and flexible suture-button constructs. Klitzman et al. [30] reported significantly greater sagittal fibular translation with suture-button fixation compared to tricortical screw fixation, supporting the decreased mechanical constraint associated with flexible constructs. Similarly, Soin et al. [31] observed broadly comparable fibular displacement between both constructs under cyclic loading, although screw fixation provided higher torsional failure strength. However, those prior studies were conducted on cadaveric specimens under controlled axial and torsional loading, whereas the present investigation utilized a synthetic physical model without externally applied axial compression, focusing specifically on relative changes in tibiotalar voltage distribution during passive sagittal motion. Therefore, the present results should be interpreted as qualitative mechanical trends characteristic of the physical simulator rather than direct representations of in vivo loading conditions.

The observations across the four evaluated experimental conditions demonstrate that the custom sensing matrix—utilizing a low-cost Velostat™ piezoresistive substrate interfaced with an ESP32 microcontroller—presents a viable method for qualitative estimation of intra-articular contact distribution, even across highly incongruent and doubly curved anatomical geometries such as the tibiotalar joint. Although the present experimental system was evaluated using a 3D-printed synthetic joint model, the architecture could be adapted for biomechanical investigations on cadaveric specimens, albeit with increased setup complexity.

The pressure-dependent electrical resistance of Velostat™ is highly non-linear [33], necessitating rigorous calibration to yield absolute quantitative stress measurements. Although multiple calibration protocols were attempted, a reliable quantitative transfer function could not be established due to the methodological constraints detailed previously. Nevertheless, the primary objective of this study—serving as a proof-of-concept to evaluate the efficacy of a cost-effective sensing apparatus in detecting qualitative spatial shifts in joint contact topology across distinct clinical states—was successfully achieved through direct comparative pattern analysis.

For the comparative analysis of distinct clinical scenarios, the implementation of a simplified polymeric articular replica combined with a streamlined loading apparatus enabled the successful evaluation of two competing surgical techniques. This physical modeling strategy circumvented the requirement for cadaveric specimens—which entail substantial logistical complexity, acquisition constraints, and inherent inter-specimen biological variability—while avoiding the overhead of complex multi-axis force-actuation systems.

However, translation of these experimental findings to real clinical scenarios must be approached with caution, as the physical simulator captures only selected mechanical attributes of the intact human ankle. Articular cartilage was modeled using a simplified compliant material layer, which does not replicate the full viscoelastic, strain-rate-dependent, heterogeneous, and biphasic behavior of native hyaline cartilage. Similarly, ligaments and adjacent soft-tissue envelopes were replaced by synthetic surrogate materials configured to approximate only isolated stiffness characteristics. Active muscle forces were omitted, and motion was applied passively; consequently, the mapped voltage distributions primarily reflect the structural interaction between articular surface geometry and passive ligamentous constraints. Furthermore, no physiological axial load representative of weight-bearing gait was applied. Because the study was conducted using a single synthetic anatomical template, natural inter-subject anatomical variability was not represented. Accordingly, the observed discrepancies should be interpreted as comparative mechanical trends intrinsic to this benchtop experimental framework rather than direct extrapolations to in vivo joint contact pressures, long-term clinical outcomes, or patient-specific biomechanics.

The experimental protocol was intentionally focused on sagittal-plane motion because dorsiflexion and plantarflexion constitute the primary degrees of freedom of the talocrural joint and represent the dominant kinematic components during human gait. Crucially, sagittal-plane motion within this physical model is not constrained to a fixed-axis hinge rotation. Instead, relative motion between the tibia and talus encompasses coupled rolling and gliding (rotation and translation) dictated by the intrinsic contours of the articular surfaces. Furthermore, the talus is not rigidly fixed to the footplate assembly, permitting subtle three-dimensional spatial realignments during articulation. Inversion–eversion and adduction–abduction are predominantly accommodated by adjacent articulations—most notably the subtalar joint—and thus exert a secondary influence on the primary tibiotalar contact mechanics evaluated herein. Nonetheless, multi-planar coupled kinematics was not explicitly reproduced in this iteration, and their biomechanical influence remains an avenue for future developments of the physical simulator.

## 5. Conclusions

The integration of a low-cost Velostat™ piezoresistive substrate with accessible microcontrollers, such as the ESP32, provides a viable solution for qualitative visualization of intra-articular contact distributions, even across complex articular surfaces characterized by high double curvature. However, the physical matrix architecture utilizing cylindrical conductive wiring significantly complicates quantitative sensor calibration due to high localized stress concentrations induced within the highly viscoelastic Velostat™ film.

Under the specific constraints of the present physical simulator, flexible stabilization showed different tibiotalar joint contact compared to rigid trans-syndesmotic screw fixation. This discrepancy was particularly pronounced during plantarflexion, where progressive diastasis of the distal tibiofibular syndesmosis and subsequent loss of tibiotalar contact were observed. Further validation is required before biomechanical or clinical comparisons can be made.

## Figures and Tables

**Figure 1:**
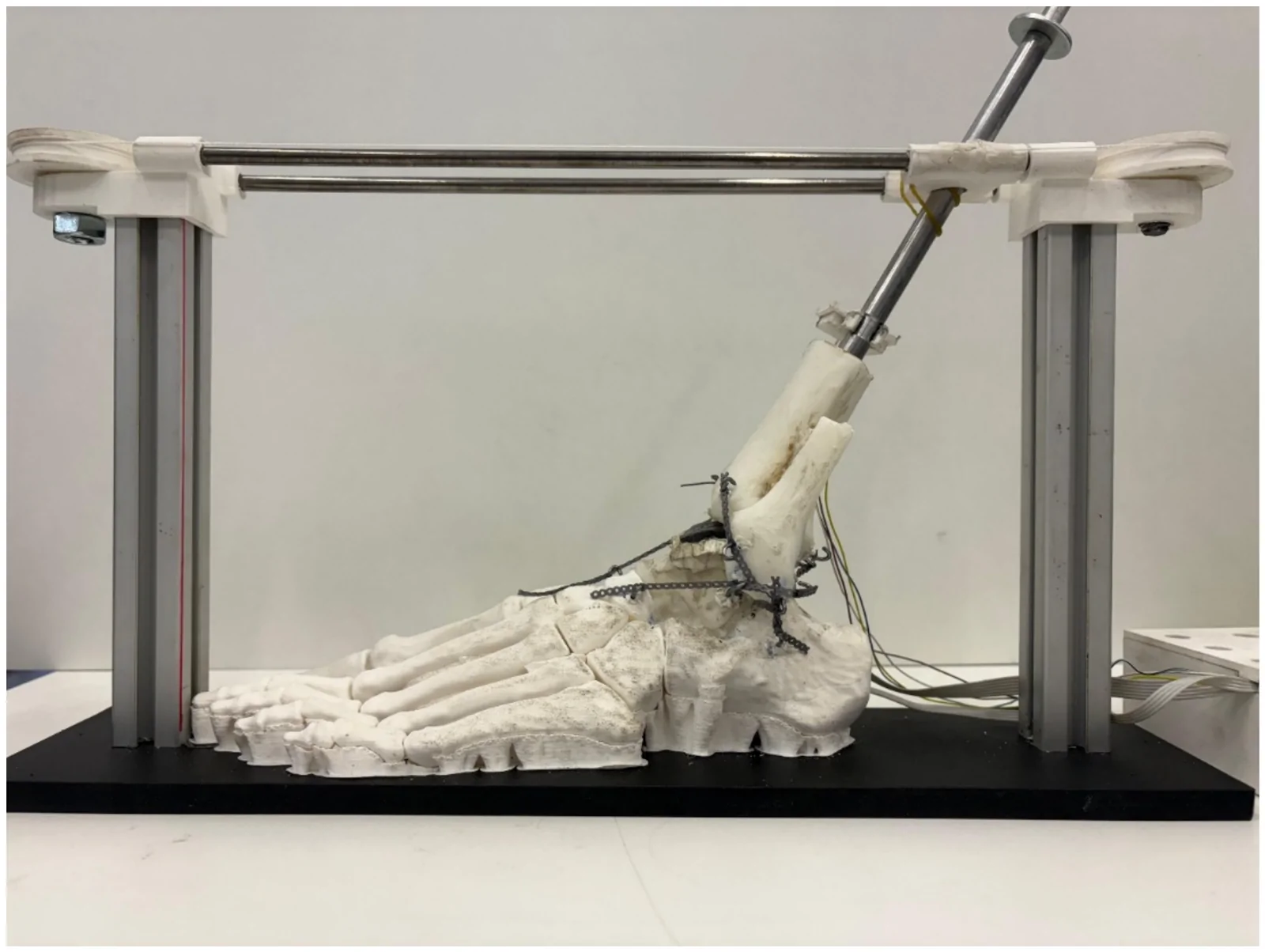
Simplified model used for the test, including the load frame.

**Figure 2:**
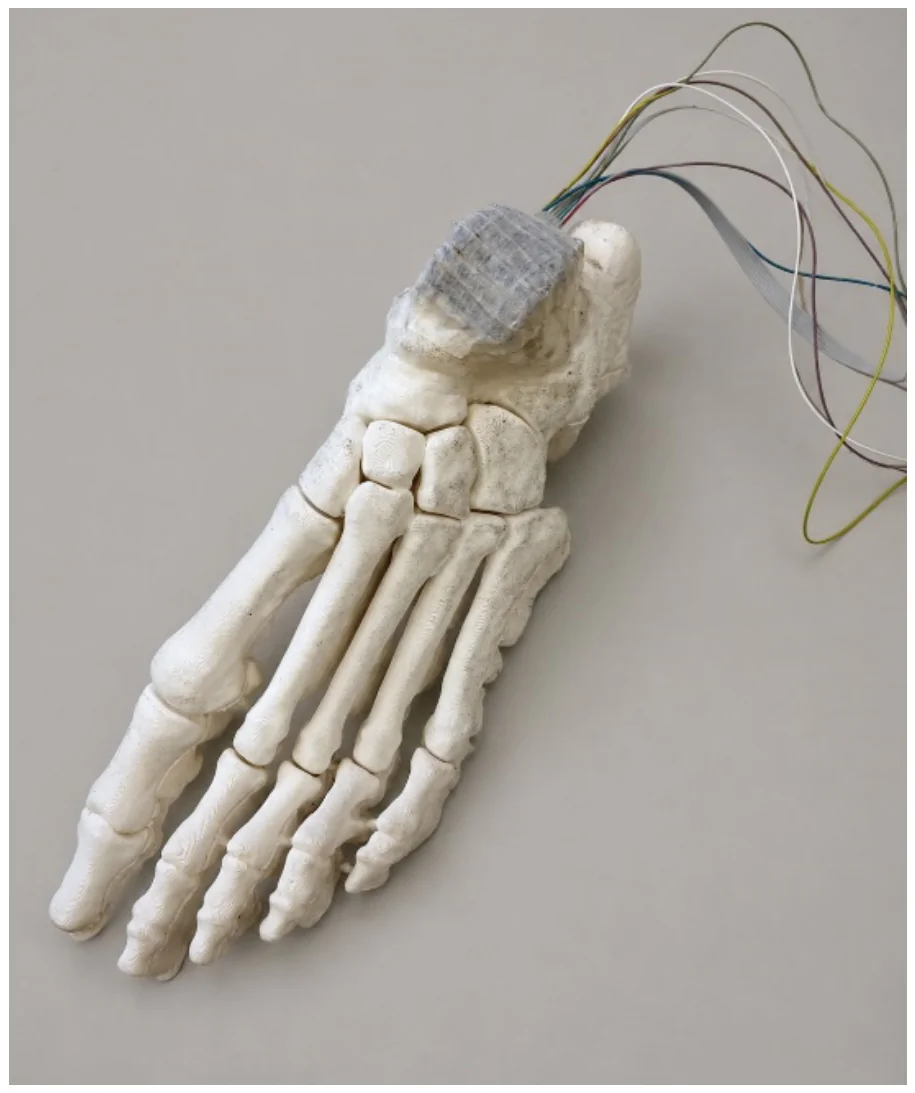
Sensing matrix on the talus.

**Figure 3:**
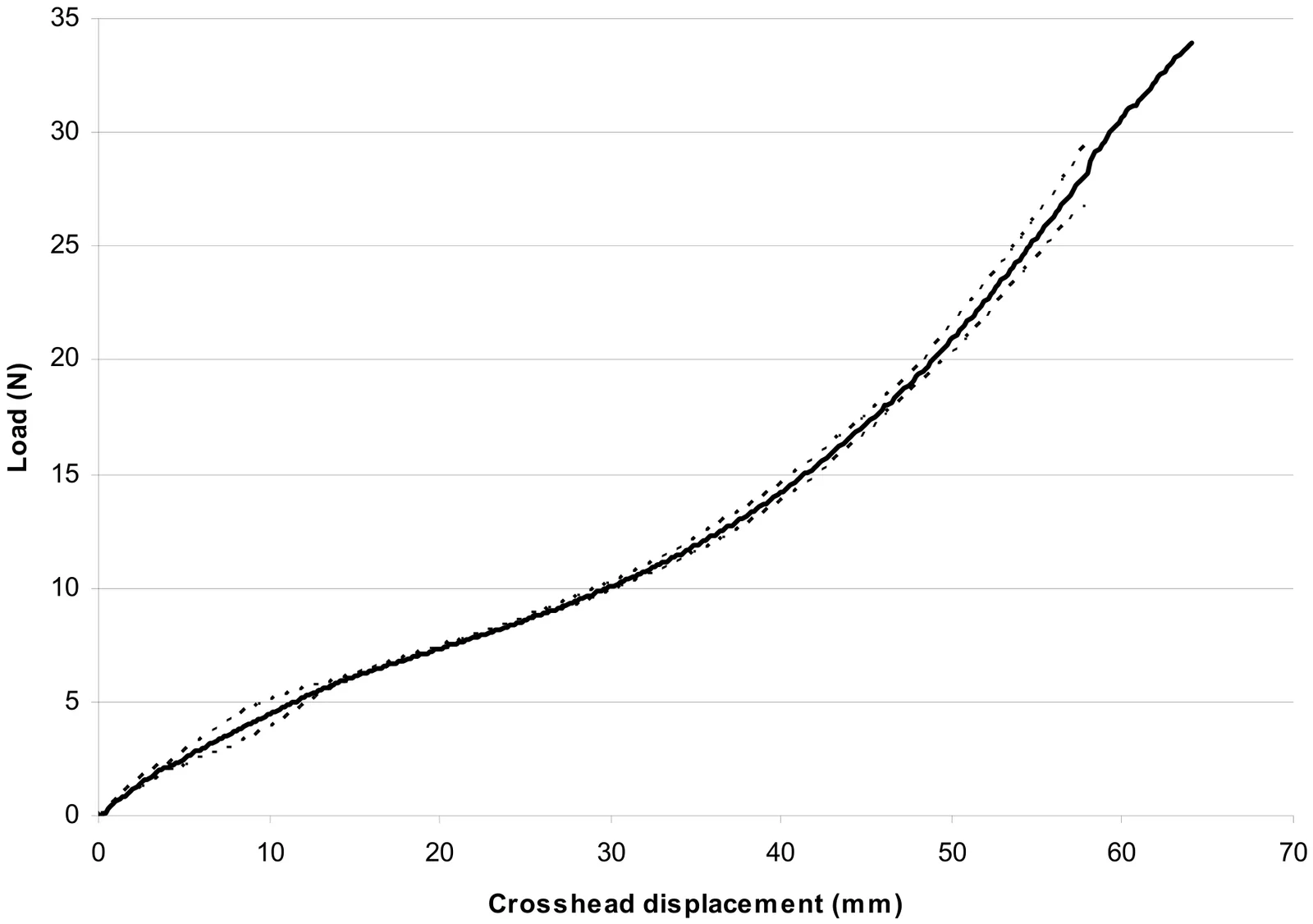
Tensile test curve of the elastic orthodontic chains.

**Figure 4:**
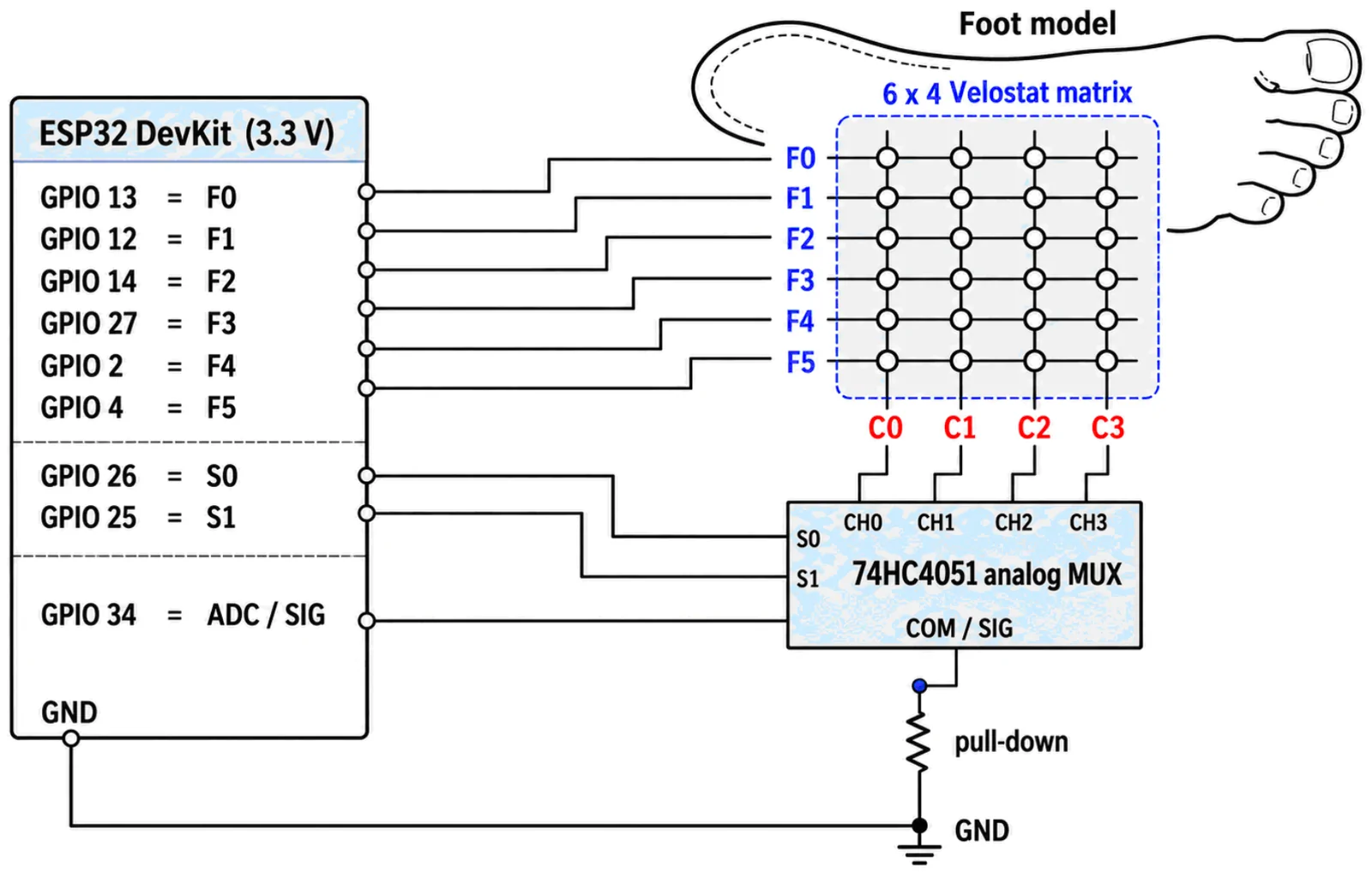
Hardware layout.

**Figure 5:**
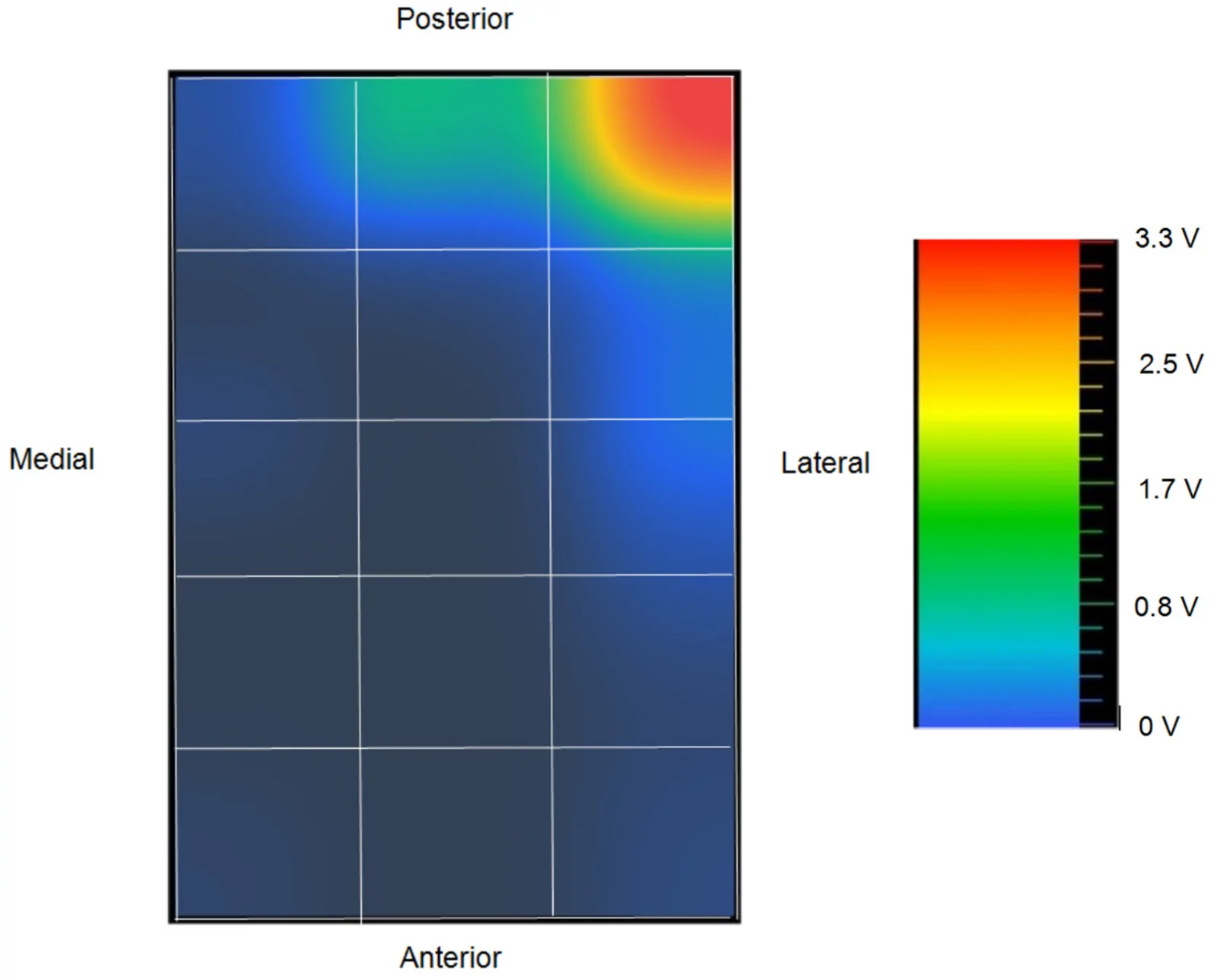
Graphical output of the voltage signal.

**Figure 6:**
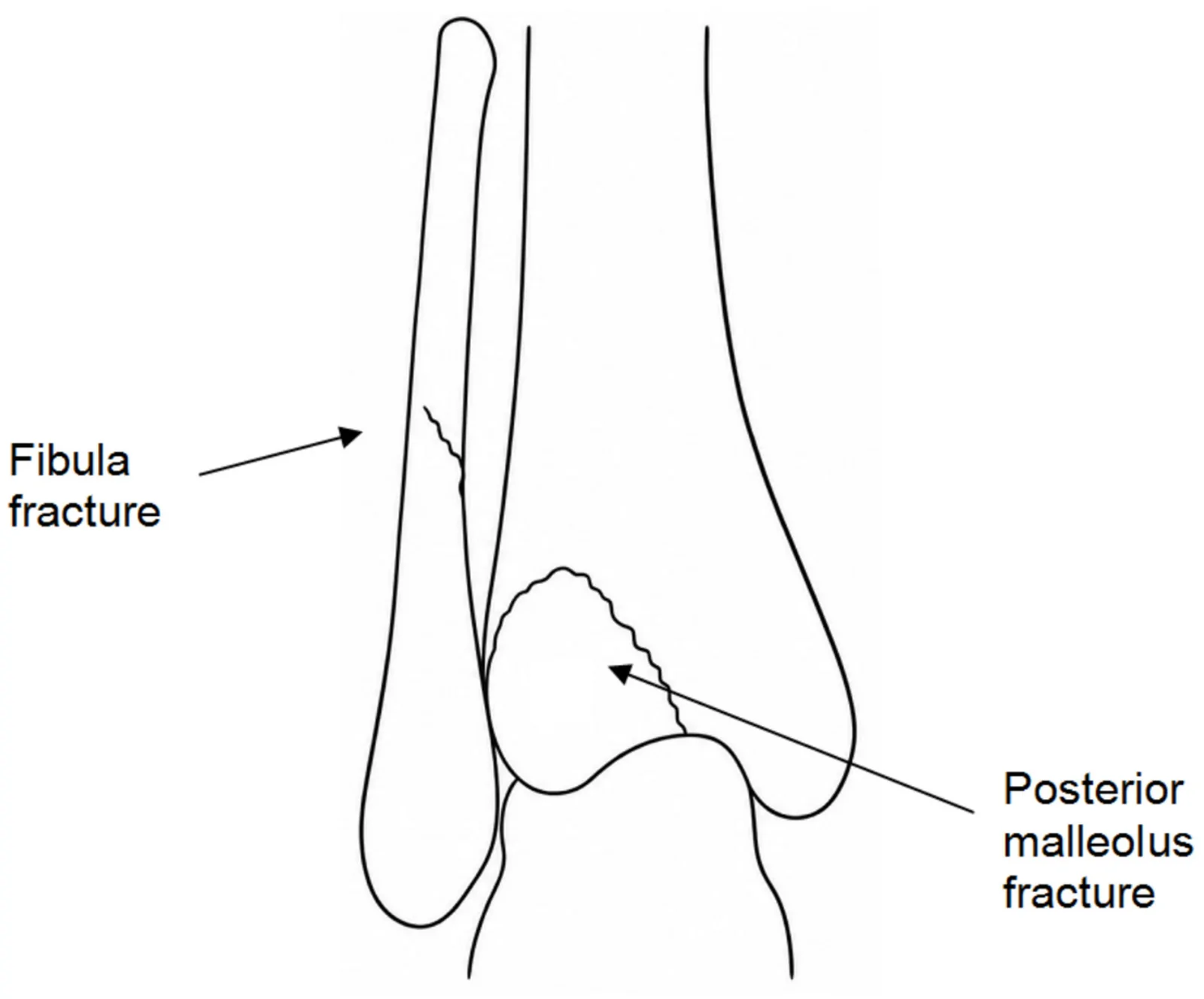
Fracture 2A from Mason.

**Figure 7:**
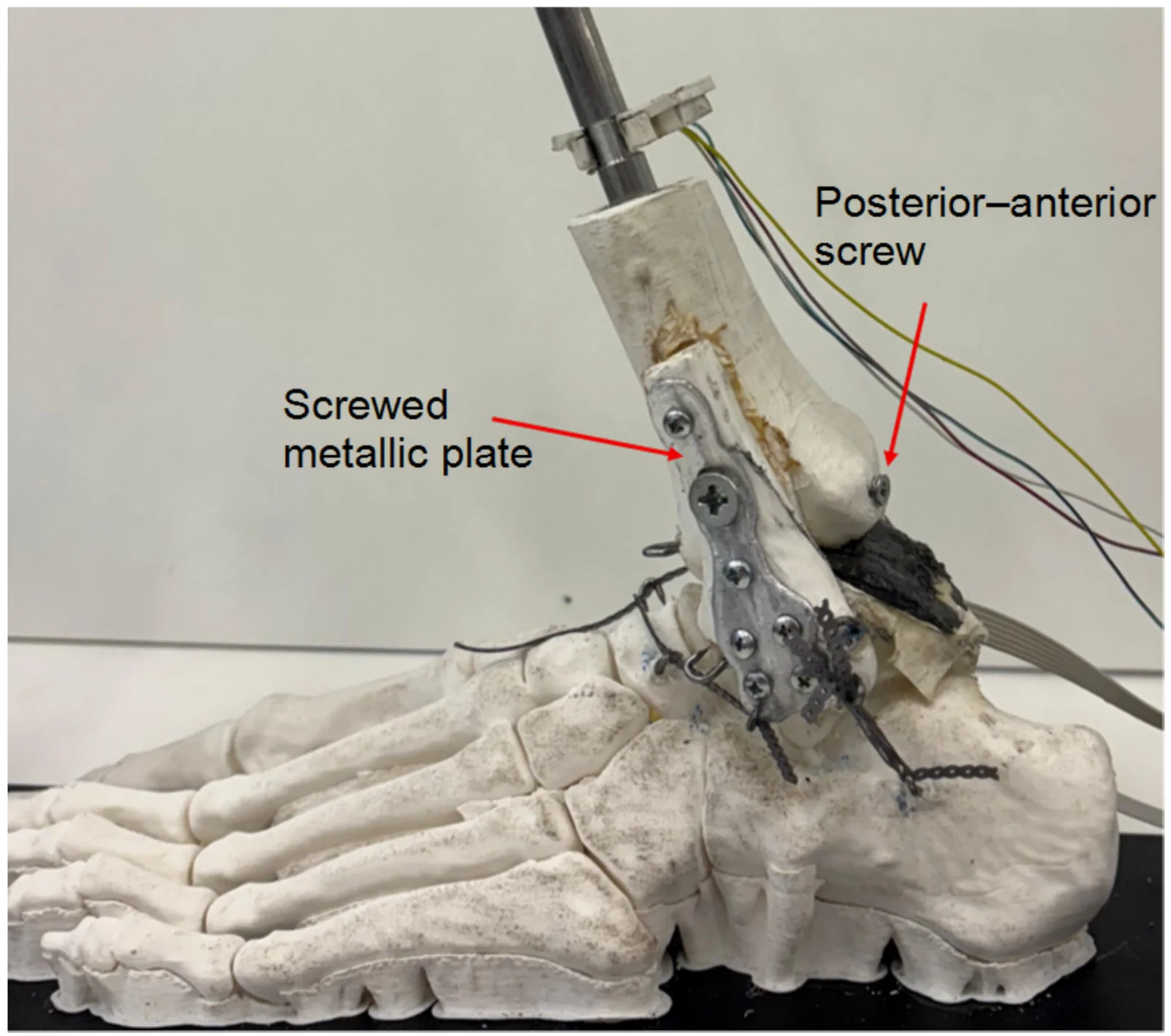
Details of fracture fixation.

**Figure 8:**
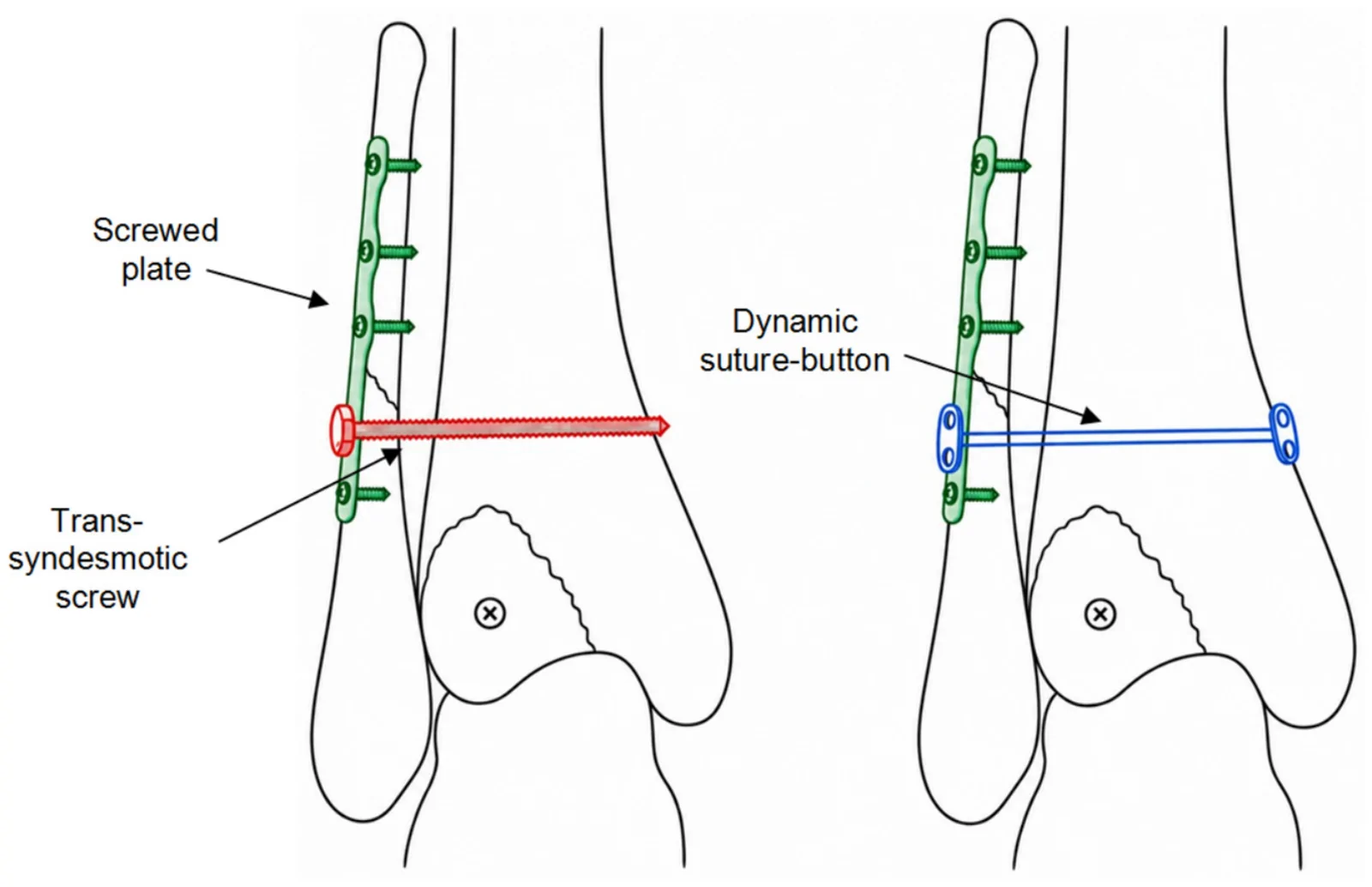
Methods of stabilization of the tibiofibular interosseous membrane. (**Left**) Trans-syndesmotic screw. (**Right**) Dynamic suture-button.

**Figure 9:**
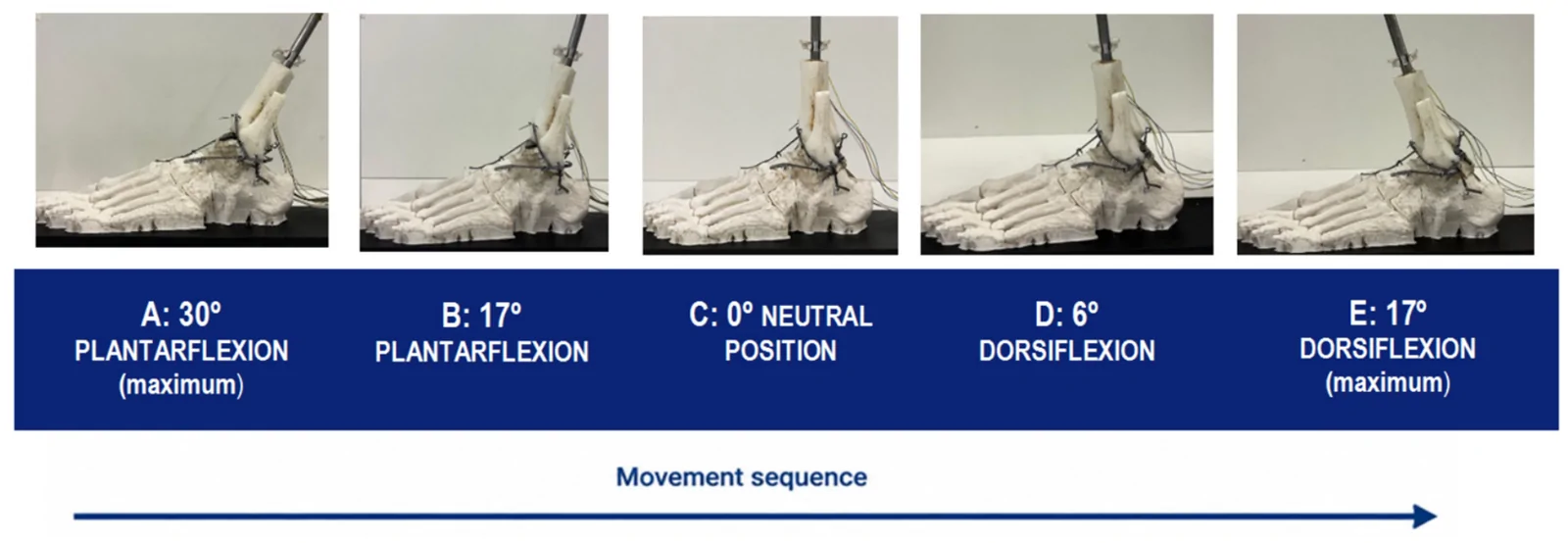
Scheme of the five positions of the carried-out tests.

**Figure 10:**
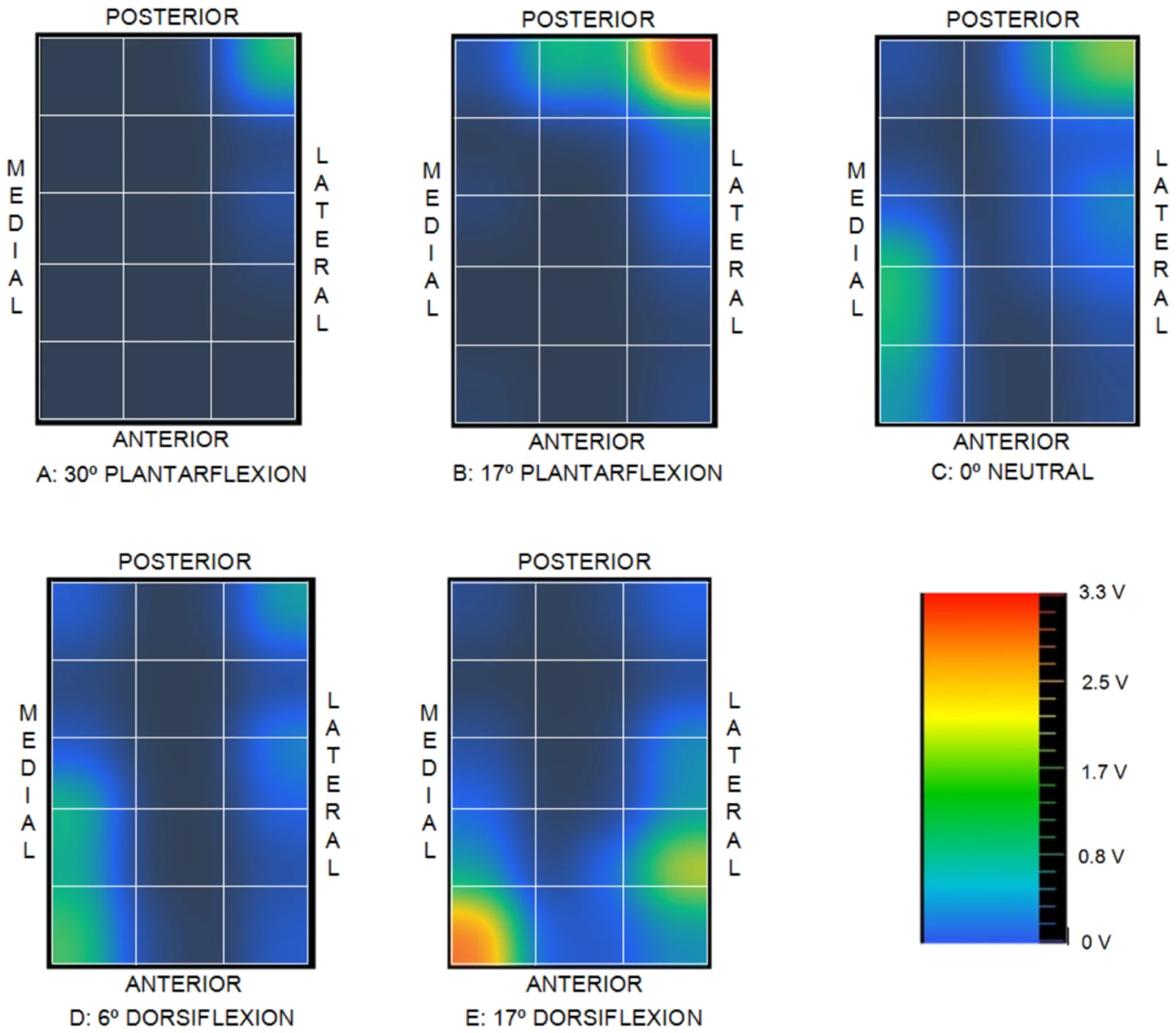
Contact maps for the intact joint, case (1).

**Figure 11:**
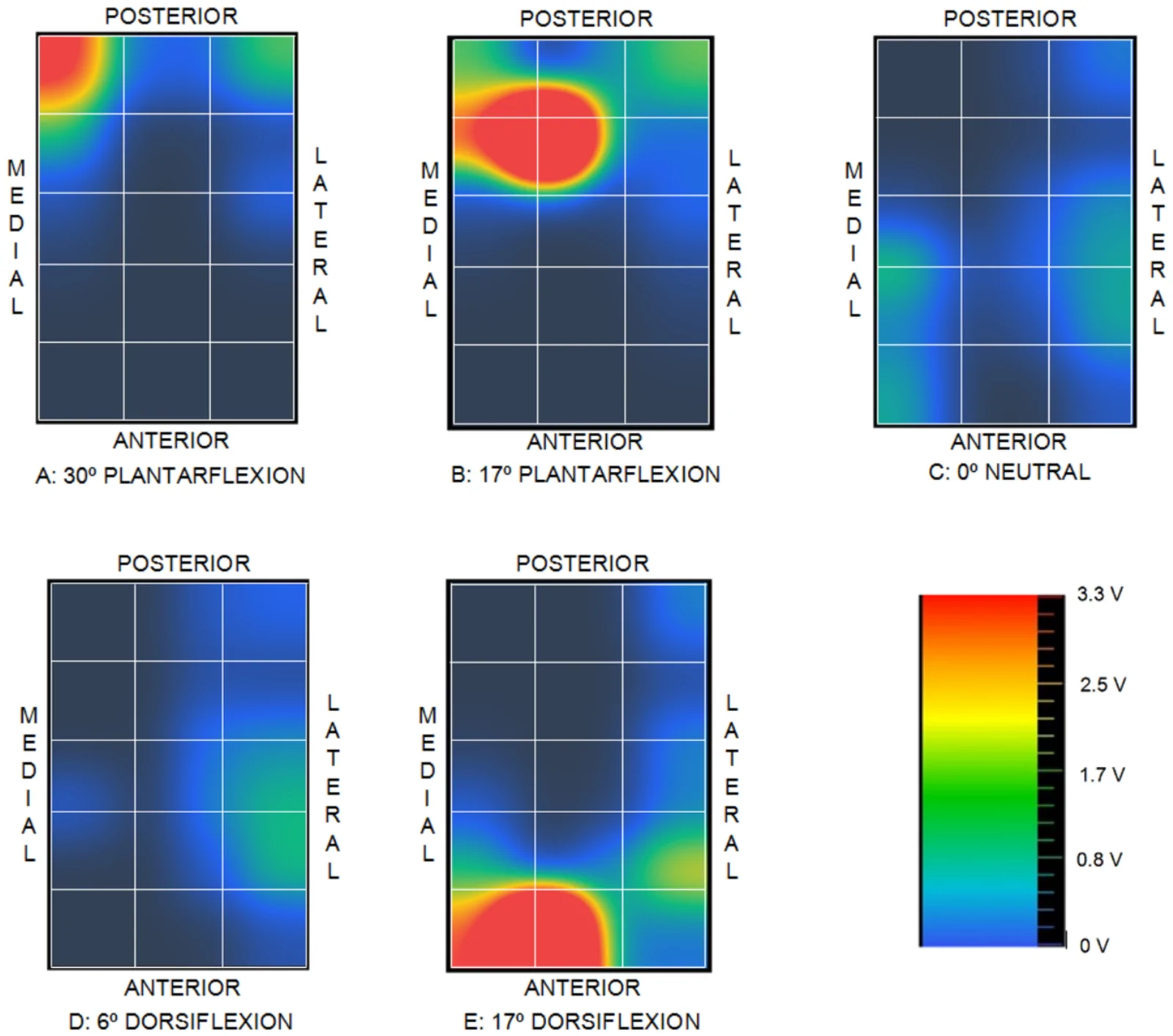
Contact maps for fractured joint, case (2).

**Figure 12:**
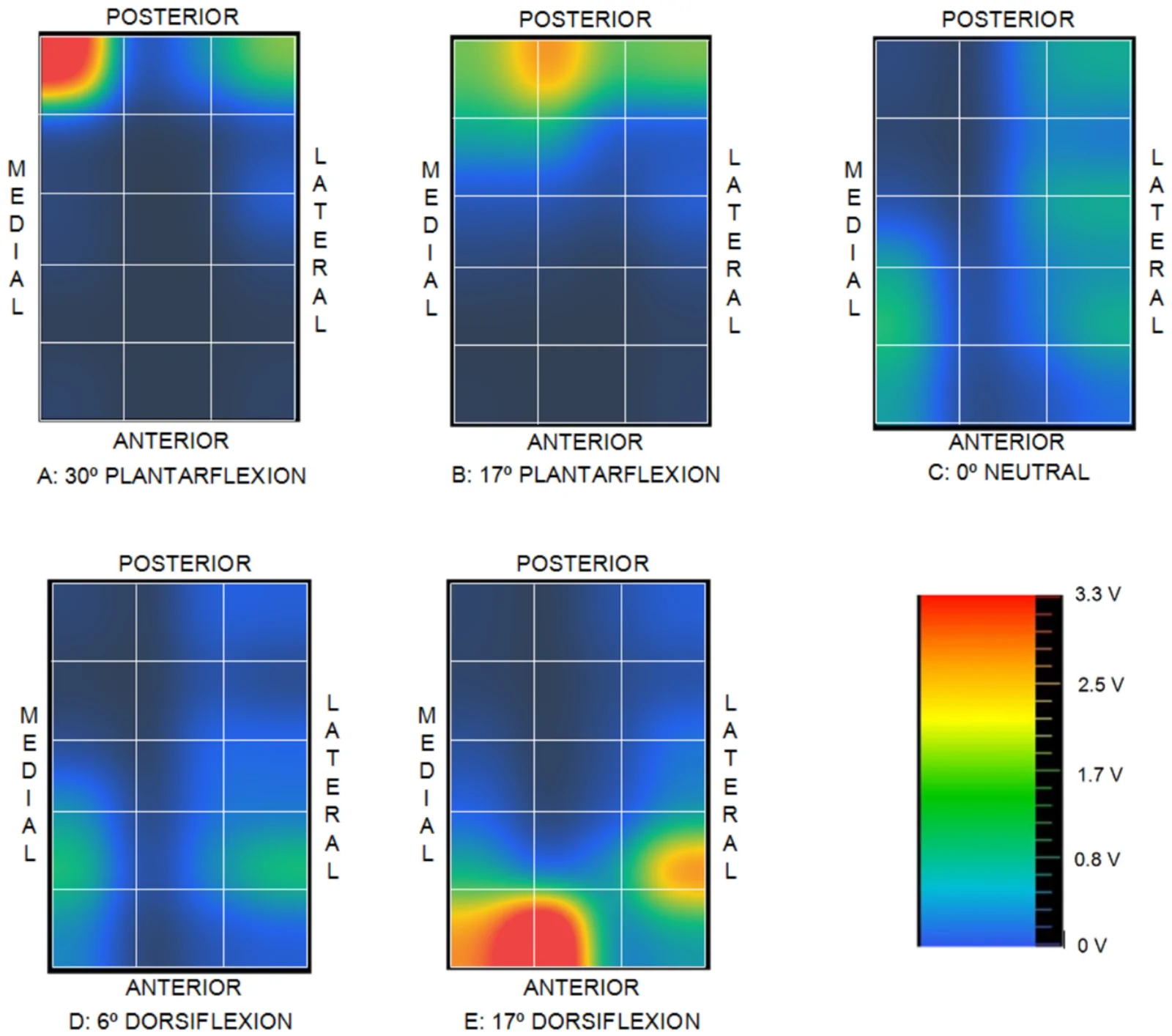
Contact maps for the distal tibiofibular syndesmosis stabilized by means of a trans-syndesmotic screw, case (3).

**Figure 13:**
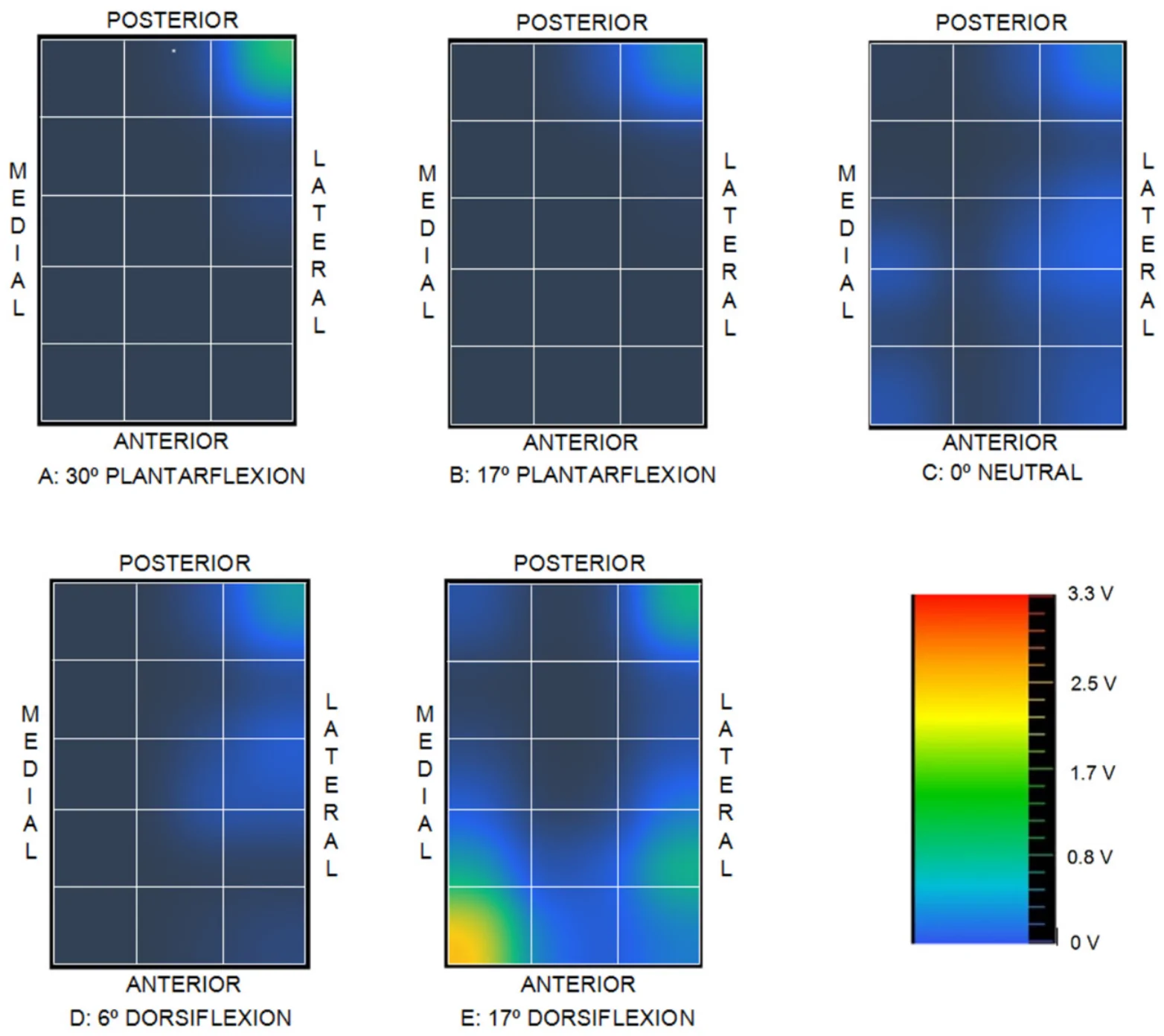
Contact maps for the distal tibiofibular syndesmosis stabilized by means of a dynamic suture-button system, case (4).

**Figure 14:**
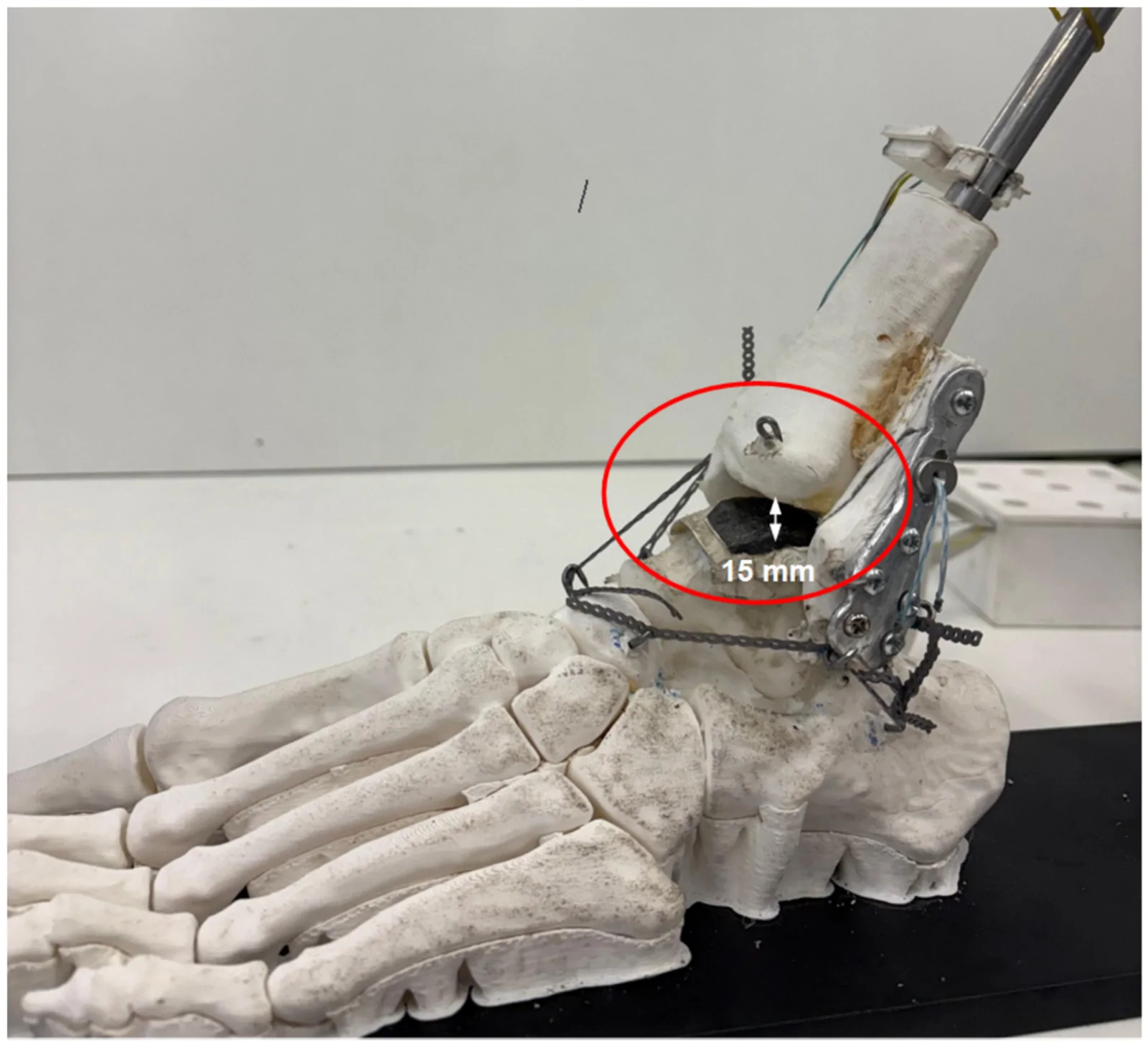
Gap between tibia and talus for case (4).

## Data Availability

Data related to this work are available upon reasonable request to the corresponding author.
